# Supplementary material for: SARS-CoV-2 Antibody Effectiveness Is Influenced by Non-Epitope Mutation/Binding-Induced Denaturation of the Epitope 3D Architecture
Source: Pathogens. 2022 Nov 29;11(12):1437. doi: 10.3390/pathogens11121437 (PMC9787365; doi:10.3390/pathogens11121437)
Supplement: Supplementary file 1 [file pathogens-11-01437-s001.zip › pathogens-2022462-supplementary.pdf]

Supplementary data

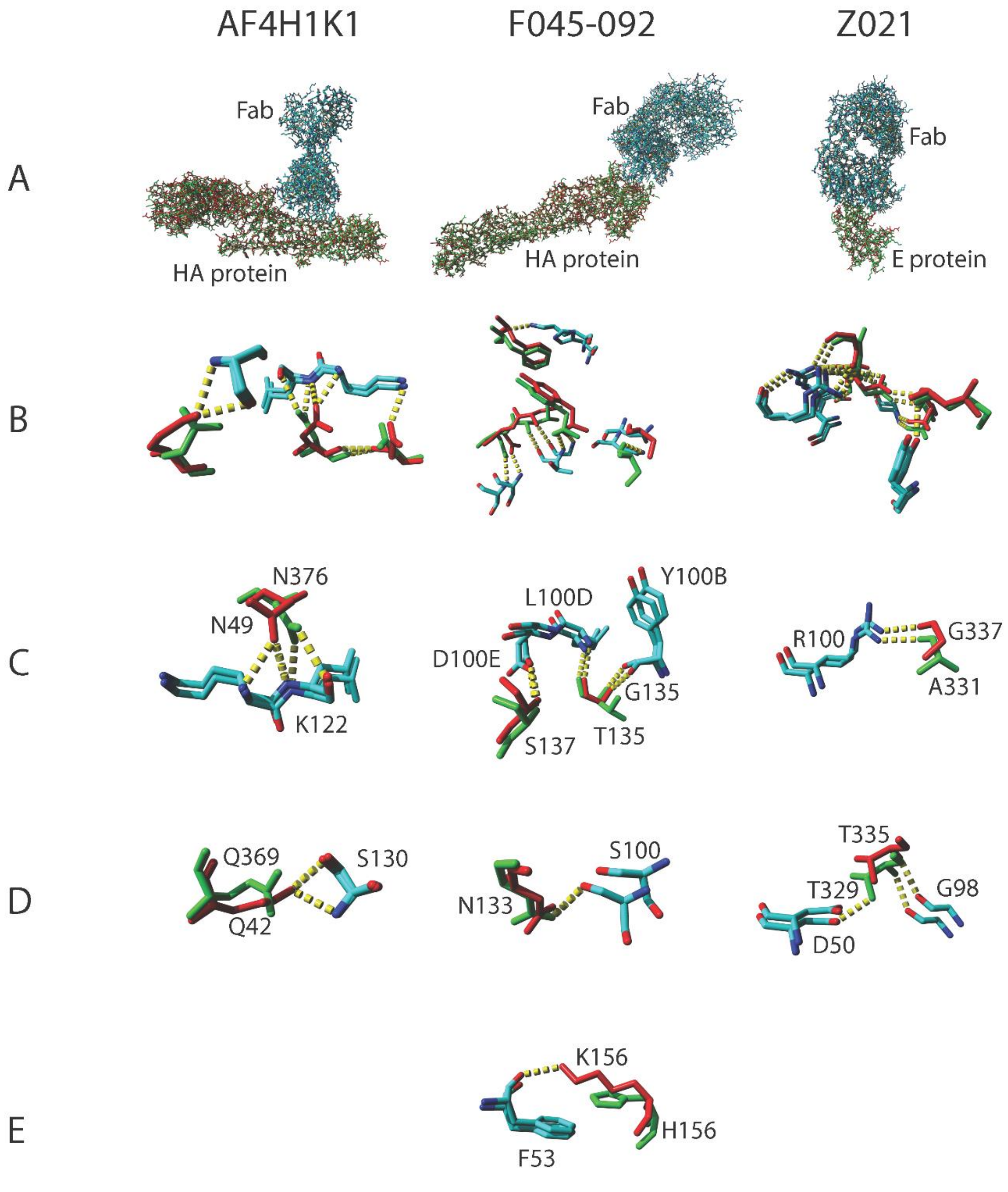

Figure S1. **Interaction of AF4H1K1, F045-092 antibodies with variant hemagglutinin, and Z021 with envelope proteins for dengue serotype-1 (DENV-1) and Zika (ZIKV) viruses.** Structural alignment of influenza H3N2 (red sticks) with H4N6 (green sticks) hemagglutinin proteins and in complex with AF4H1K1 and H3N2-A/Victoria/361/2011 (green sticks) with H3N2-A/Victoria/3/1975 (red sticks) hemagglutinin proteins in complex with F045-092, and DENV-1 (green sticks) with ZIKV (red sticks) envelope proteins in complex with Z021 (A). Comparison of the structural positioning of corresponding epitope amino acids in two different structurally aligned viral proteins (B – E). Red boxes and dotted yellow lines represent the paratope-epitope interface and hydrogen bonds, respectively. HA – hemagglutinin, E – envelope, Gly – Glycine, and Ala – Alanine.

A

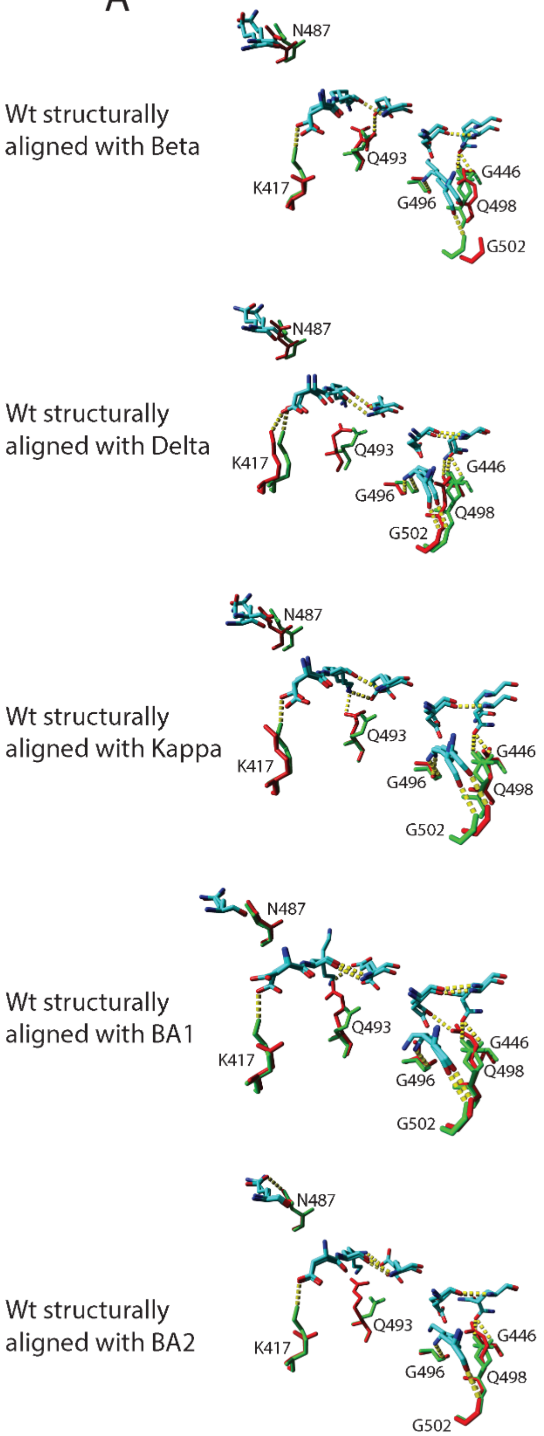

B

ACE2	WT						
	K417	G446	N487	Q493	G496	Q498	G502
Q24			5.3				
D30	2.9						
K31				4.2			
E35				3.9			
D38							
Q42		4.2				2.6	
K353					3.2		2.8

ACE2	BETA						
	K417[N]	G446	N487	Q493	G496	Q498	G502
Q24			3.5				
D30							
K31				6.2			
E35				2.8			
D38							
Q42		4.3				4.5	
K353					4.7		4.2

ACE2	DELTA						
	K417	G446	N487	Q493	G496	Q498	G502
Q24			3.1				
D30	2.8						
K31				2.7			
E35				5.6			
D38							
Q42		3.1				4.2	
K353					2.7		2.5

ACE2	KAPPA						
	K417	G446	N487	Q493	G496	Q498	G502
Q24			3.2				
D30	4.7						
K31				2.8			
E35				2.4			
D38							
Q42		4.4				3.9	
K353					2.5		3.1

ACE2	OMICRON – BA.1						
	K417[N]	G446[S]	N487	Q493[R]	G496[S]	Q498[R]	G502
Q24			2.8				
D30							
K31				3.1			
E35							
D38						2.9	
Q42							
K353							2.8

ACE2	OMICRON – BA.2						
	K417[N]	G446	N487	Q493[R]	G496[S]	Q498[R]	G502
Q24			2.6				
D30							
K31				4.6			
E35							
D38						3.6	
Q42		4.1					
K353							3.1

Figure S2. **Interaction of ACE2 with SARS-CoV-2 variants of concern (VOC).** Comparison of the structural positioning of corresponding epitope amino acids in the structurally aligned wild-type RBD protein (green sticks) with the Beta; Delta; Kappa; BA1; and BA2 RBD proteins (red sticks) (A). Distance in Angstroms between acceptor/donor atoms on ACE2 interacting amino acids and the same complementary donor/acceptor atoms on epitope representative amino acids on structurally aligned original and VOC RBD proteins (B). The dotted yellow lines and [] represent the hydrogen bonds and substituted amino acids in VOC RBD, respectively.

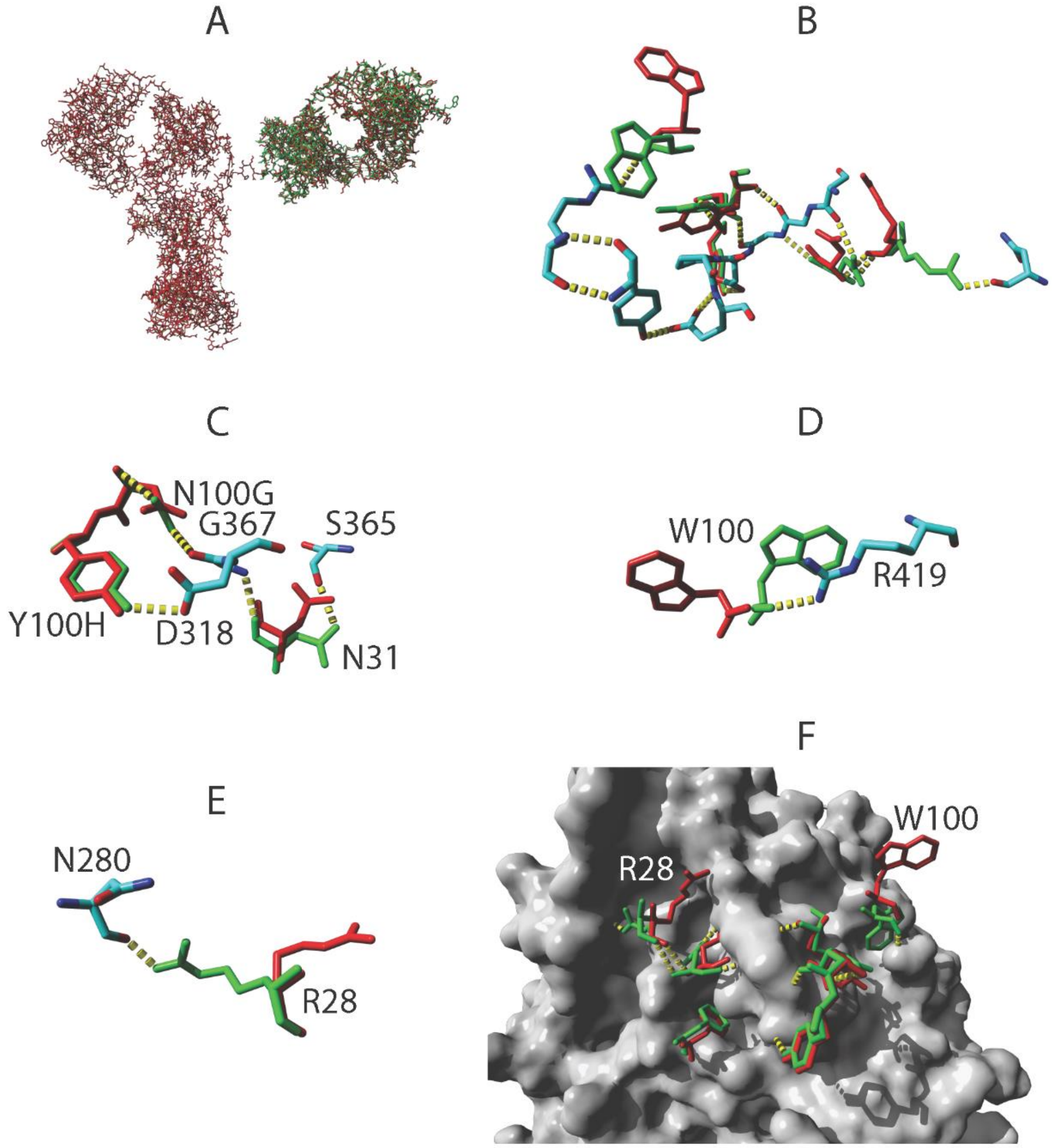
A
B
C
N100G
G367
S365
Y100H
D318
N31
D
W100
R419
E
N280
R28
F
W100
R28

Figure S3. **The structural positioning of paratope AASCs in IgG versus Fab.** Structural alignment of a b12 Fab (green sticks) with its parent IgG (red sticks) (A). Flashed out paratope amino acids in structurally aligned IgG (red sticks) with its Fab (green sticks) in complex with HIV gp120 (element sticks) (B – E). Surface (gray) representation of HIV gp120 in complex with structurally aligned b12 Fab (green sticks) and its parent IgG (red sticks) (F). Hydrogen bonds are depicted as dotted yellow lines.

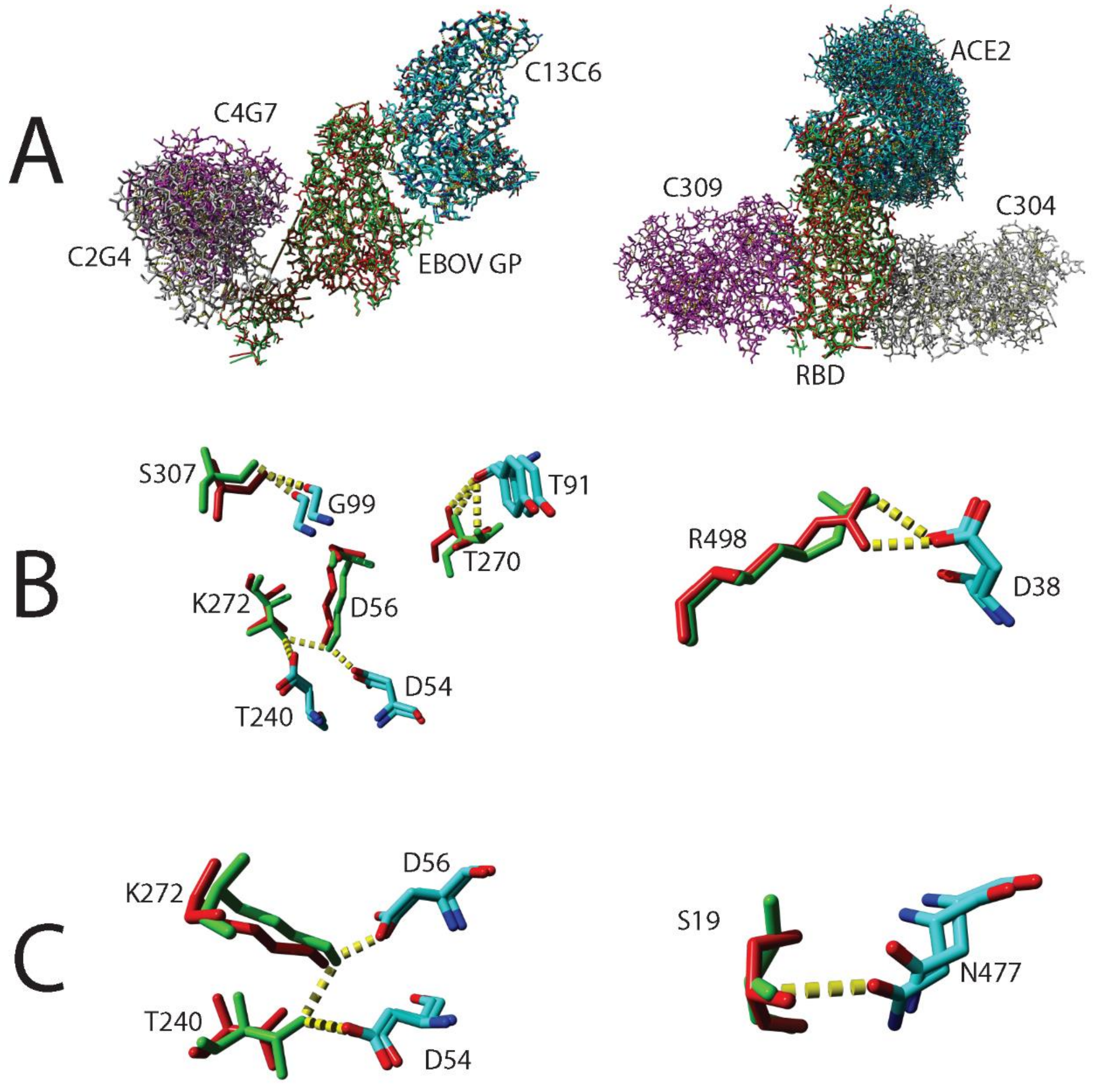
A
C4G7
C13C6
C2G4
EBOV GP
ACE2
C309
C304
RBD
B
S307
G99
T91
T270
K272
D56
D54
T240
R498
D38
C
K272
D56
T240
D54
S19
N477

Figure S4. **Differential binding of C13C6 and ACE2 to Ebola virus glycoprotein (EBOV GP) and RBD, respectively, when used individually or as antibody/ACE cocktails.** Structural alignment of the same antigens in complex with individual antibodies/ACE2 or a cocktail of antibodies (A). Exposure of selected paratope-epitope AASCs with EBOV GP proteins presented as red and green sticks while antibodies are depicted as element, gray, and magenta sticks (B). Exposure of selected paratope-epitope AASCs with RBD proteins presented as red (antibody/ACE2 cocktail) and green (individual ACE2) sticks while antibodies/ACE2 are depicted as element, gray, and magenta sticks (C). Hydrogen bonds are presented as dotted yellow lines.

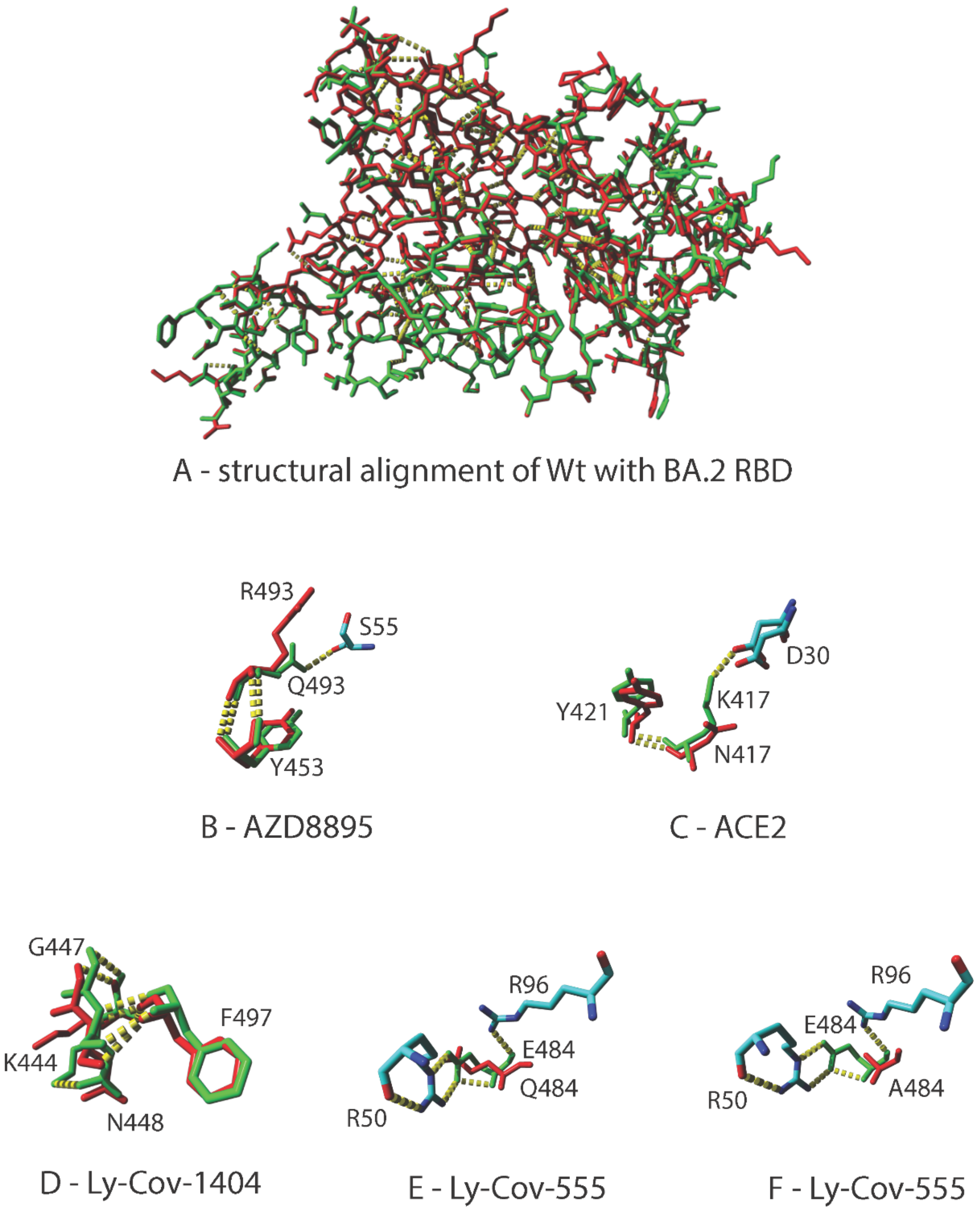
A - structural alignment of Wt with BA.2 RBD
R493
S55
Q493
Y453
B - AZD8895
D30
K417
Y421
N417
C - ACE2
G447
F497
K444
N448
D - Ly-Cov-1404
R96
E484
Q484
R50
E - Ly-Cov-555
R96
E484
R50
A484
F - Ly-Cov-555

Figure S5. **Factors influencing the substitution of amino acids under immune pressure.** Structurally aligned the original SARS-CoV-2 (element sticks) and BA.2 (gray sticks) RBD proteins highlight the crucial role of hydrogen bonds in stabilizing proteins (A). Flashed out corresponding amino acids of structurally aligned SARS-CoV-2 wild-type and BA.2 RBD proteins (element sticks) in complex with ADZ8895 (B), ACE2 protein (C), LY-CoV-1404 (D), and LY-CoV-555 (E and F) antibodies (green sticks). Hydrogen bonds are presented as dotted yellow lines.

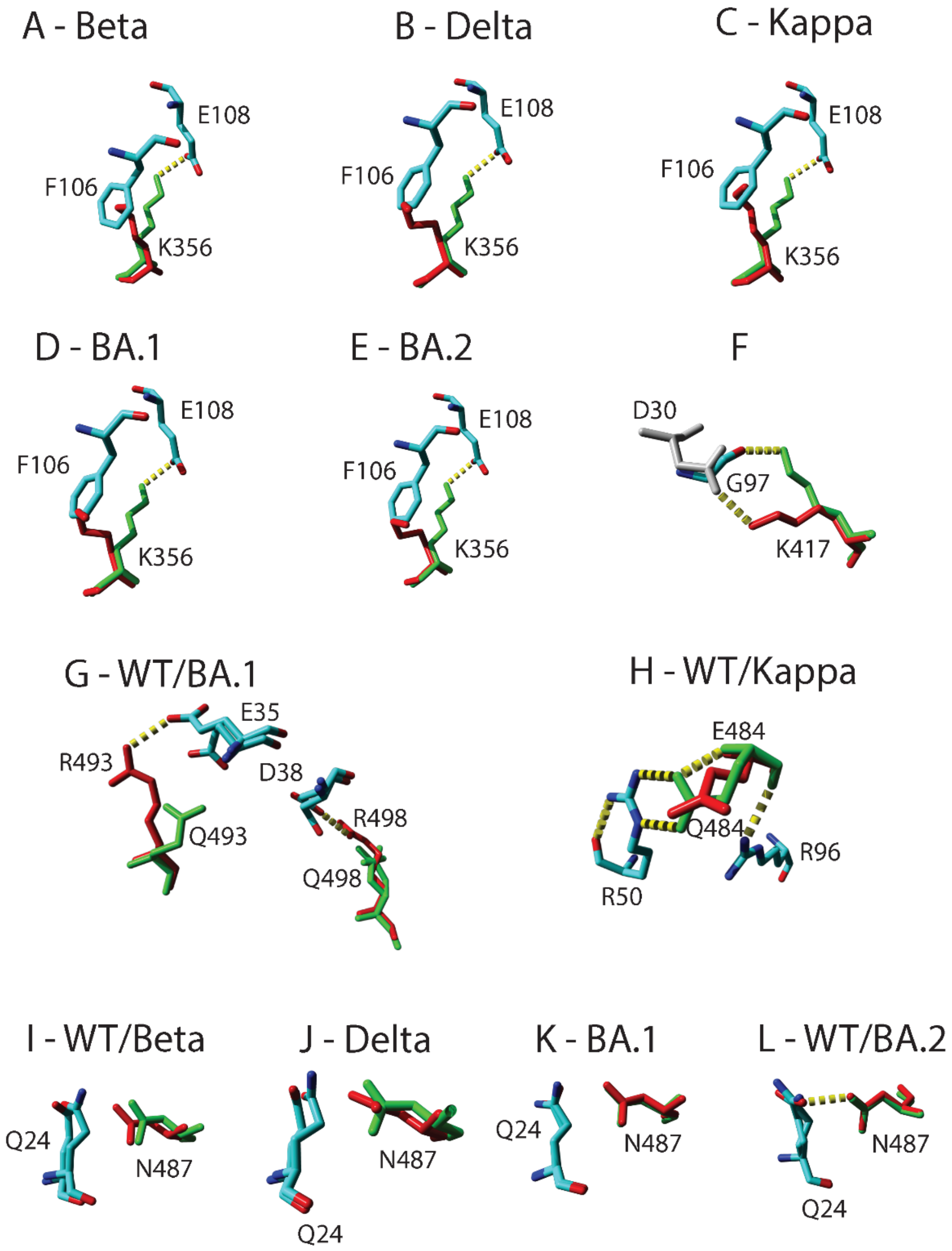

M

ACE2	N487				
	WT	BETA	DELTA	BA.1	BA.2
Q24	5.3	3.5	3.1	2.8	2.6

Figure S6. Mutation-driven mechanisms are used by SARS-CoV-2 VOC to escape neutralizing antibody recognition while improving ACE2 binding. Flashed out AASCs of the structurally aligned the original RBD with various VOC RBD proteins (green and red sticks) complexed with antibodies S309 (A – E) and C102 (F) (element sticks) and ACE2 (G) (gray sticks). Replacement of donor/acceptor atoms on structurally aligned the original and Kappa epitope AASCs presented as element sticks in complex with an LY-CoV-555 antibody (H). Qualitative and quantitative SARS-CoV-2 (N487) in complex with ACE2 (Q24) are presented as green/red and element sticks (I – L) and Table (M). Hydrogen bonds are presented as dotted yellow lines. WT – wild-type.
